# Novel Approaches for Assessing Circadian Rhythmicity in Humans: A Review

**DOI:** 10.1177/0748730420940483

**Published:** 2020-07-23

**Authors:** Derk-Jan Dijk, Jeanne F. Duffy

**Affiliations:** * Surrey Sleep Research Centre, Faculty of Health and Medical Sciences, University of Surrey, Guildford, UK; † UK Dementia Research Institute, University of Surrey; ‡ Division of Sleep and Circadian Disorders, Departments of Medicine and Neurology, Brigham and Women’s Hospital, Boston, Massachusetts, USA; § Division of Sleep Medicine, Harvard Medical School, Boston, Massachusetts, USA

**Keywords:** biomarkers, machine learning, transcriptomics, data science, mathematical models, light, heart rate, skin temperature

## Abstract

The temporal organization of molecular and physiological processes is driven by environmental and behavioral cycles as well as by self-sustained molecular circadian oscillators. Quantification of phase, amplitude, period, and disruption of circadian oscillators is essential for understanding their contribution to sleep-wake disorders, social jet lag, interindividual differences in entrainment, and the development of chrono-therapeutics. Traditionally, assessment of the human circadian system, and the output of the SCN in particular, has required collection of long time series of univariate markers such as melatonin or core body temperature. Data were collected in specialized laboratory protocols designed to control for environmental and behavioral influences on rhythmicity. These protocols are time-consuming, expensive, and not practical for assessing circadian status in patients or in participants in epidemiologic studies. Novel approaches for assessment of circadian parameters of the SCN or peripheral oscillators have been developed. They are based on machine learning or mathematical model-informed analyses of features extracted from 1 or a few samples of high-dimensional data, such as transcriptomes, metabolomes, long-term simultaneous recording of activity, light exposure, skin temperature, and heart rate or *in vitro* approaches. Here, we review whether these approaches successfully quantify parameters of central and peripheral circadian oscillators as indexed by gold standard markers. Although several approaches perform well under entrained conditions when sleep occurs at night, the methods either perform worse in other conditions such as shift work or they have not been assessed under any conditions other than entrainment and thus we do not yet know how robust they are. Novel approaches for the assessment of circadian parameters hold promise for circadian medicine, chrono-therapeutics, and chrono-epidemiology. There remains a need to validate these approaches against gold standard markers, in individuals of all sexes and ages, in patient populations, and, in particular, under conditions in which behavioral cycles are displaced.

Assessing the phase, period, and amplitude of circadian oscillators is central to the study of circadian rhythms, be it in cyanobacteria, mice, or humans (Kuhlman et al., 2018). Accurate phase measurements enable description of the phase relationship (relative timing) of circadian oscillations with the environmental and behavioral cycles in the study of entrainment in humans (Duffy et al., 1999; Wright et al., 2005). Accurate phase assessments are also a prerequisite to describe the interrelations between circadian oscillators in any multioscillator system, be it hierarchically organized or not (Honma, 2018; Dijk and Lockley, 2002). Accurate assessments of period and amplitude enable identification of mechanisms underlying abnormal entrainment or lack of robustness in circadian regulation of physiological processes. Developing accurate and unobtrusive methods to assess period, phase, amplitude, robustness, and disruption is critical in understanding the role of circadian rhythms in physical and mental health and their disorders. Traditional human circadian rhythm research areas include shift work, jet lag, and circadian rhythm sleep-wake disorders (Sack et al., 2007a). More recently, phenomena such as social jet lag and applications like chrono-therapeutics and chrono-medicine have gained attention, and the epidemiology of circadian disruption is an emerging area of interest (Roenneberg and Merrow, 2016). Methods that can accurately assess circadian parameters and be implemented at scale and at low cost are critical for the translation of basic circadian rhythm research to all these areas (Cederroth et al., 2019; Mullington et al., 2016; Munch and Kramer, 2019). Recent years have seen the introduction of novel approaches to the assessment of circadian parameters and, in particular, circadian phase in humans. Some of these methodologies use machine-learning approaches to extract features that predict circadian parameters from high-dimensional “omics” data, whereas others are based on multiple behavioral, environmental, and physiological variables collected from research or consumer-grade wearables, combinations of mathematical modeling and wearable-based data acquisition, or analyses of *in vitro* circadian behavior in human cell cultures. Here, we will revisit (Duffy and Dijk, 2002) some of the issues, pitfalls, and requirements for the assessment of circadian parameters in humans and discuss some of the novel approaches within that context.

## Diurnal Rhythmicity, Circadian Rhythmicity, Endogenous Circadian Components, behavioral masking

Rhythmicity may be observed in any physiologic or behavioral variable, and quantification of this rhythmicity may be of intrinsic interest. However, rhythmicity is often assessed not for its own sake but rather to quantify characteristics of an underlying circadian oscillator, which is assumed to drive the rhythmicity being assessed. A central concept in circadian rhythm research is that an overt 24-h diurnal rhythm can only be considered a circadian rhythm when it persists in the absence of masking by external environmental and behavioral 24-h cycles (Kuhlman et al., 2018). Many laboratory experiments in which organisms, including humans, were studied while shielded from 24-h environmental cycles have demonstrated that at least part of overt rhythmicity is driven by endogenous circadian oscillators. However, those studies have also revealed that 24-h environmental and behavioral cycles contribute to 24-h rhythmicity in many aspects of physiology. This distinction between diurnal rhythmicity (due to 24-h rhythms in the environment and in behavior) and circadian rhythmicity (due to endogenous processes) is critical to take into account when developing and applying methods for the quantitative assessment of circadian rhythmicity. Unfortunately, this important distinction is often overlooked, and we believe it has hampered progress in developing novel methods for assessing circadian timing and in understanding the role of circadian oscillators in disease (Lyall et al., 2018).

In the context of the distinction between diurnal and circadian rhythmicity, human circadian rhythm researchers also make a distinction between endogenous circadian components of a rhythm and evoked components. This distinction relates to the concept of behavioral masking and implies that assessment of the endogenous circadian component of a rhythm not only requires that components driven (masked) by environmental cycles are removed but also that components driven (masked) by behavioral cycles must be controlled (Rietveld et al., 1993). It has now become clear that behavioral and environmental masking extends to rhythmicity at the molecular level. Thus, rhythmicity in the brain and blood transcriptome is to a large extent driven by the timing of the sleep-wake cycle (Maret et al., 2007; Hor et al., 2019; Archer et al., 2014; Archer and Oster, 2015) and is affected by insufficient sleep (Moller-Levet et al., 2013; Laing et al., 2019b). Likewise, rhythmicity in the liver is to a large extent driven by feeding rhythms and influenced by light (Atger et al., 2015; Greenwell et al., 2019; Koronowski et al., 2019). Protocols such as the constant routine or forced desynchrony, which eliminate or distribute masking uniformly across the circadian cycle, were developed to control this behavioral masking so that aspects of rhythmicity driven “directly” by circadian clocks could be quantified. These protocols have been used primarily in human studies, and the confounding effects of behavioral cycles, and the rest-activity cycle in particular, have often been ignored in animal studies.

Novel circadian biomarkers must be robust to altered environmental and behavioral influences for them to be useful in patients with circadian rhythm disorders, in people who do shift work or have recently travelled to another time zone, and in individuals with social jet lag or who keep irregular sleep schedules. Therefore, in our evaluation of novel methods to quantify circadian rhythmicity, we consider the extent to which they can distinguish between environmental, behavioral, or endogenous circadian components of overt rhythmicity.

## A biomarker for circadian phase, amplitude, and period of which oscillator?

**Figure 1.**
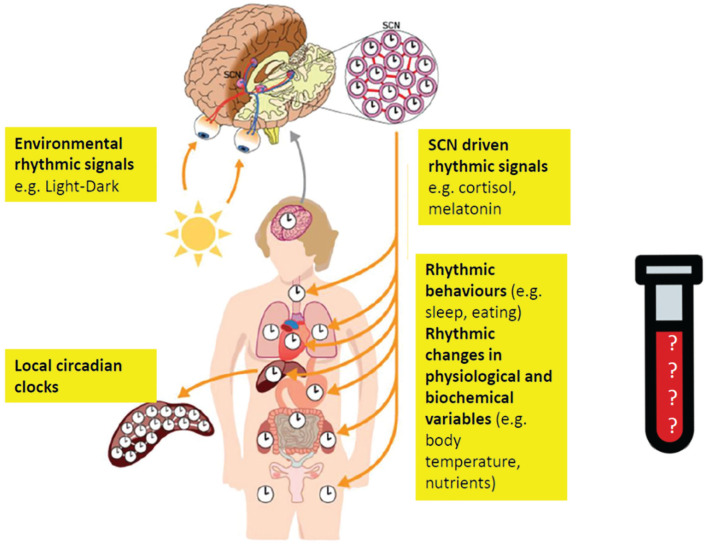
A biomarker for which rhythm and what generates this rhythm? Modified from Bollinger and Schibler (2014). Structure of the human circadian timing system. Molecular clocks and circadian rhythms are present in the brain, including the SCN-based oscillator and periphery. A circadian biomarker may provide information about the rhythms in the SCN or in peripheral tissues and organs. Rhythms in organs and tissues are influenced by external rhythmic signals, SCN-driven signals, local circadian oscillators, and behavioral rhythms, such as sleep or eating. A biological sample will contain many features (transcripts, proteins, metabolites). These various features will be influenced by SCN input, the local circadian oscillator, and behavior. The selection of the final feature set for the biomarker will depend on the purpose of the biomarker (e.g., assessing SCN phase or phase of tissue-specific circadian oscillator). In many cases, the tissue or organ of interest will not be accessible, and the features will be extracted from, for example, blood, which makes the identification of robust biomarkers even more challenging.

Now that it has been established that circadian oscillators are present in every tissue, organ, and cell, it is more important than ever to be explicit about the circadian oscillator of interest when assessing a rhythmic output (Mohawk et al., 2012; Mure et al., 2018; Fig. 1). Traditionally, and particularly so in research on circadian rhythm sleep-wake disorders, there has been a focus on assessing parameters of the master circadian pacemaker located in the SCN (Sack et al., 2007a, 2007b). Obviously, there is more to the human circadian system than the SCN. Cardiologists may be interested in the phase, period, or amplitude of circadian oscillators in the heart (Thosar et al., 2018) or how the timing of antihypertensives affect blood pressure control (Smolensky et al., 2017), and clinicians or researchers of metabolism may want to assess circadian parameters in the pancreas or adipocytes (Qian and Scheer, 2016). In fact, insulin sensitivity rhythms, glucose rhythms, and many other rhythms related to metabolism have already been characterized in humans (Poggiogalle et al., 2018). Immunologists may be interested in the extent to which the various white blood cell types vary across the diurnal cycle (Pick et al., 2019), how molecular processes related to immune function oscillate within each of these cell types, and how far these intracellular or intercellular rhythms are directly modulated by local circadian clocks (Baxter and Ray, 2019; Downton et al., 2019). Oncologists are interested in rhythmicity in tumors and how circadian phase assessment may allow the most effective timing of chemo- or radiotherapies (Shafi and Knudsen, 2019; Shuboni-Mulligan et al., 2019). Neurologists and psychiatrists are interested in circadian rhythms in mood, seizures, and neurodegeneration (Logan and McClung, 2019; Khan et al., 2018; Leng et al., 2019; Pavlova et al., 2009; Lucey et al., 2017). Whether these rhythms in heart rate, glucose, insulin sensitivity, or leukocytes are driven by local tissue clocks and/or through central control from the SCN, and to what extent these rhythms reflect “endogenous” circadian rhythms, or diurnal rhythmicity, or instead are driven by rhythmic behaviors, is not easily established. Nevertheless, novel methods and biomarkers to quantify rhythmicity and biomarkers may facilitate the characterization of tissue-specific local oscillators, allowing for increased understanding of many normal and pathological physiological processes and the application of chronotherapies targeting specific organs and tissues.

Standard parameters of circadian oscillators and the rhythms they generate are phase and intrinsic period, but aspects such as amplitude and waveform of rhythms can also be used to characterize rhythms, although the latter two are rarely assessed.

## Traditional Peripheral markers for SCN Phase and Period

The SCN drives many daily rhythms, including those observed in the autonomic nervous system (Buijs et al., 2013), endocrine rhythms (Czeisler and Klerman, 1999; Morris et al., 2012), and in particular the circadian component of the sleep propensity rhythm (Dijk and Czeisler, 1995). The desire to assess characteristics of the circadian pacemaker located in the SCN arose from hypotheses predicting that changes in endogenous circadian parameters were the cause of changes in sleep timing, such as observed in aging, circadian rhythm sleep-wake disorders, or between chronotypes (Sack et al., 2007b). Diagnosis and understanding of the etiology of circadian rhythm sleep-wake disorders may be informed by accurate assessment of the SCN’s intrinsic period, amplitude, and/or phase. This desire to understand the status of the SCN pacemaker is also driven by the recognition that effects of sleep-wake therapeutics, be it light treatment or melatonin administration, are dependent on the circadian phase at which they are administered (Duffy and Wright, 2005; Keijzer et al., 2014). Effective timing of chrono-therapies aiming to correct SCN-driven rhythms requires accurate assessment of the SCN phase to determine when they should be applied to obtain the desired results.

Because the SCN is not directly accessible in humans, the timing of peripheral markers is used as a proxy for SCN phase. The choice of which marker(s) to use is influenced by the ease of assessment, cost, and reliability of the marker itself. While sleep-wake propensity is influenced by the circadian system, the timing of the sleep-wake cycle is not considered a reliable marker of SCN phase or period in humans (Czeisler et al., 1999).Even though the circadian (SCN) phase at which sleep occurs affects the duration and structure of sleep, studies of shift workers and jet-lagged travelers as well as laboratory studies of spontaneous and forced desynchrony have demonstrated that sleep can occur at many phases and that the relationship between the timing of the sleep-wake cycle and SCN phase varies across conditions and both between and within individuals (Dijk and Lockley, 2002).

Peripheral rhythms such as core body temperature, cortisol, and melatonin rhythms have been used extensively as markers for circadian phase of the SCN. Several lines of evidence support the validity of these peripheral markers for SCN phase. Neuroanatomical tracer and lesion studies show that through its projections to the paraventricular nucleus and other hypothalamic nuclei, the SCN drives rhythmicity in melatonin and cortisol as well as core body temperature (Moore, 2013). Further evidence comes from the observation that exposure to light induces equivalent phase shifts in these phase markers (Czeisler et al., 1990), and the sleep-propensity rhythm is closely coupled to the core body temperature and melatonin rhythms (Dijk et al., 1997). Reductions in amplitude induced by light pulses or phase shifts of the light-dark and sleep-wake cycle are correlated across melatonin, cortisol, and core body temperature (Dijk et al., 2012; Czeisler et al., 1990).

**Figure 2.**
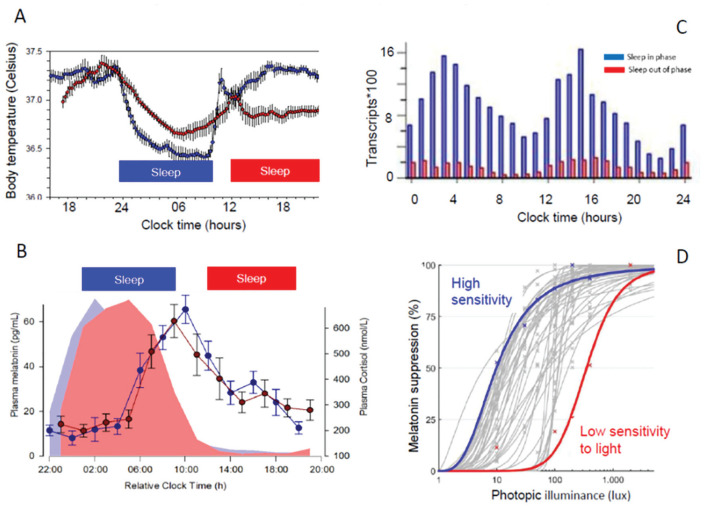
Effects of sleep-wake cycle and light exposure on rhythmic variables. (A) Daily rhythm of core body temperature is altered when sleep occurs in phase (at night) versus out of phase. Recalculated from Dijk and Czeisler (1995). (B) Rhythms of plasma melatonin and cortisol are not much affected by sleeping in phase (during the night) or out of phase (during the day). Data from Archer et al. (2014). (C) Frequency distribution of the acrohases of rhythmic transcripts in whole blood when sleeping in phase (blue) and out of phase. From Archer et al. (2014). (D) Individual-level dose-response curves for melatonin suppression and light levels. Blue, high-sensitivity individual; red, low-sensitivity individual. From Phillips et al. (2019).

Given the validity of these phase markers, assessing the phase of the SCN relative to clock time or to the external light-dark cycle, or even relative to another oscillator, would seem to be straightforward. However, the key pitfall in using these phase markers remains that their overt rhythmicity is composed of both “endogenous” circadian components and “evoked” components. In humans, the plasma melatonin rhythm serves as an example of this. In constant darkness, a prominent rhythm of melatonin concentration in blood or saliva can be observed, with high values during the biological (subjective) night and low values during the biological (subjective) day. However, light has long been known to suppress melatonin. While initially it was thought that only bright light could do so, it is now recognized that light intensities as low as 6 lux can acutely suppress melatonin by 50% in some participants, thereby “masking” the endogenous SCN phase and amplitude (Phillips et al., 2019; Zeitzer et al., 2000; Fig. 2D). Therefore, accurate estimation of the phase of the melatonin rhythm requires a time series of blood or saliva samples to be collected in very dim light (Benloucif et al., 2008), a requirement that is not easily met in the real world. The cortisol rhythm, besides being affected by light (Rahman et al., 2019), is also masked by stress and fasting but, just like melatonin, is little affected by sleep (Oster et al., 2017; Fig. 2B).

Whereas, in the case of melatonin, the primary confounding variable is environmental (i.e., light), for other markers of SCN phase, the primary confounding variables are behavioral. Core body temperature is lower at night than during the day. The amplitude and phase of the observed core body temperature rhythm are very much influenced by the timing of rest/sleep. This is demonstrated by data showing that when sleep occurs at night, the amplitude of the temperature rhythm is high, whereas when sleep occurs during the day, the amplitude of the rhythm is greatly reduced (Fig. 2A). In fact, this masking can be so extreme as to cause an observed temperature nadir to occur during the day, even though the true endogenous nadir (revealed under appropriate conditions) is located at night. The masking impact of sleep on core temperature is due to a combination of supine posture, inactivity, and sleep itself (Krauchi and Deboer, 2010). The difficulty of assessing circadian phase on the basis of temperature may be further illustrated by contrasting core temperature and skin temperature: whereas sleep lowers core temperature, skin temperature rises when we lie down and fall asleep, and the pattern of temperature varies across distal and proximal parts of the body (Krauchi et al., 2000; Krauchi and Wirz-Justice, 1994). The conditions in which assessment of SCN phase is of most interest are also conditions in which this assessment is most challenging. In shift work and jet lag, sleep will often be displaced from the normal circadian phase and/or normal clock time. Altered phase relationships between sleep and SCN phase also occur in circadian rhythm sleep-wake disorders, social jet lag, and neurodegeneration (Rahman et al., 2009). Furthermore, the sleep-wake cycle is almost always associated with cycles of dark-light and fasting-feeding, and these latter cycles also affect physiological, endocrine, and molecular rhythms. Thus, these peripheral markers (melatonin, cortisol, core temperature) can be used to assess SCN status only when the masking effects of environmental and behavioral cycles are adequately controlled. For the same reasons, adequate environmental and behavioral control should be a prerequisite in the search for any novel methods to assess SCN status.

Univariate markers such as melatonin, cortisol, or core temperature can only be used as markers of the SCN status by collection of a time series. The time series should be sufficiently long to identify the phase (i.e., minimum, maximum, onset or offset, of the variable under study). If the amplitude of the central clock is of interest, the time series must be at least a full circadian cycle (24 h). In the search for novel methods to assess SCN status, a minimum duration time series of at least 24 h should be used.

In summary, peripheral univariate markers can be used to assess circadian parameters such as phase and amplitude, but they require carefully controlled conditions, costly and labor-intensive protocols, with repeated sampling of blood, urine, or saliva over extended periods of time, and are often burdensome for the research participant.

The dim-light melatonin onset (DLMO; Lewy et al., 1999) assessed in blood or saliva has emerged as the gold standard marker of choice, but urinary 6-sulphatoxy melatonin has also been used successfully (e.g., Lockley et al., 2015), although the duration of collection is longer and the temporal resolution of the phase assessment may be lower than for the DLMO.

## DLMO as a biomarker for SCN Phase

The term *biomarker* is frequently used in medicine and clinical trials and is defined as “a characteristic that is objectively measured and evaluated as an indicator of normal biological processes, pathogenic processes, or pharmacologic responses to a therapeutic intervention” (Menetski et al., 2019). Some of the required or desirable characteristics of a biomarker are that it is present in easily accessible tissues or fluids, that it can be reliably quantified through established essays, and that it is sensitive, specific, and valid in a wide variety of situations (robustness) and populations. The DLMO is essentially a biomarker for SCN phase, and it meets many biomarker requirements. Importantly, even though melatonin is masked by light, the masking effects of the sleep-wake cycle itself on melatonin are small. This implies that melatonin is a robust marker for central circadian phase in those conditions in which the phase relationship between sleep and SCN may be changed.

Even though the DLMO is considered a gold standard, its assessment is not without error. This might be simple measurement error, such as assay errors or errors related to imperfect implementation of the dim-light protocol. In addition, it may be that the phase relation between the melatonin rhythm and the relevant SCN rhythm (e.g., the phase-response curve to light or the sleep-propensity rhythm) varies between individuals or conditions.

Kronauer and colleagues (2002) estimated the error of melatonin-based phase assessments of the human circadian pacemaker in a detailed comparison of the variability of phase assessments using melatonin, cortisol, and core body temperature. It was concluded that melatonin-based methods were superior and that the standard deviation for melatonin-based methods ranged from 14 to 21 min. This implies that within an individual, phase differences of more than an approximately 30-min phase can be reliably assessed. For a description of how accuracy and uncertainty of melatonin-based phase assessments are affected by sampling frequency, analysis methods, populations studied, and thresholds applied, we refer to Klerman et al. (2002), Danilenko et al. (2014), and Lewy et al. (1999).

In its standard implementation, the main drawbacks of the DLMO are that (1) it requires special environmental conditions (dim light) and (2) it requires a time series of at least several hours of samples. The required sampling frequency and duration of the time series depends on any a priori knowledge about the approximate phase and on the required precision of the phase assessment. In many situations, it can be assumed that melatonin will rise sometime in the evening hours or early night (a few hours prior to usual bedtime), whereas in other situations, no such assumptions can be made (e.g., non–24-h sleep-wake disorder, shift work, or jet lag), and a time series of 24 h may be required to capture the DLMO. Whereas the DLMO is most commonly assessed under controlled laboratory conditions, protocols for the use of the DLMO in the home environment have been developed and validated in patient populations (Keijzer et al., 2011; Pullman et al., 2012; Burgess et al., 2016).

## Requirements/Desirables for Novel Approaches fo assess Parameters of the SCN in Humans

The main aim of novel approaches is to overcome the limitations of current approaches. At the same time, these novel approaches should meet the requirements outlined above, such as being robust against masking effects and be able to accurately reflect SCN phase. Thus, novel phase markers should be evaluated against a gold standard phase marker for SCN (e.g., DLMO). The precision of the biomarker should meet the requirements of its application or use case. Importantly, for many applications/use cases, the biomarker should assess the circadian parameter of interest with sufficient precision to quantify the parameter at the level of the individual, rather than the “group” level.

### How to Quantify the Performance of a New Marker?

The performance of a new marker against a gold standard can be quantified in a variety of ways, and different publications use different methods. Some of the metrics used are the error and its standard deviation, the absolute error and its standard deviation, the median error and its range, the fraction of samples with an error less than a particular threshold, the correlation between gold standard and novel marker, regression analysis of gold standard versus novel method, and there are probably others. All of these methods have their advantages and disadvantages, and their validity depends on whether underlying assumptions about the distribution of the data (e.g., a normal distribution) are valid. An issue relevant for assessing errors of phase is that the circular nature of the data needs to be taken into account (e.g., a phase at 23:59 and 00:02 h are very close). Another point to keep in mind is that a mean error may simply represent a systematic bias and can be corrected for, and an absolute error is useful only if a systematic error (bias) cannot be corrected for.

## Novel Approaches to assess Circadian Phase in Humans

**Figure 3.**
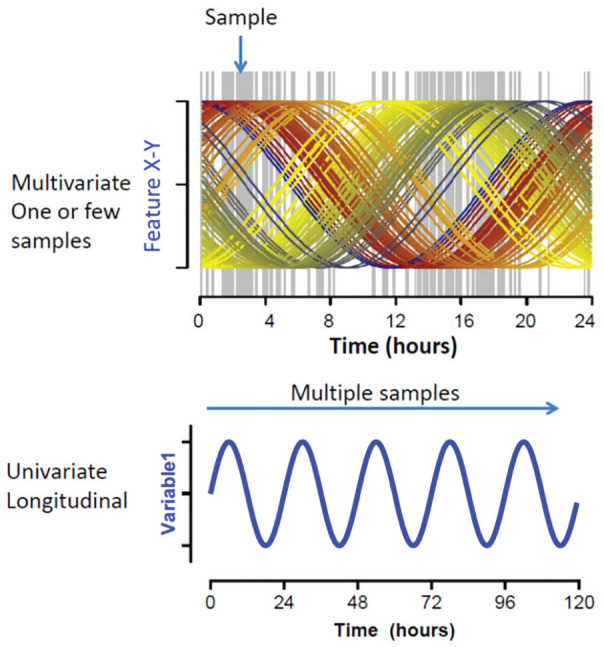
Univariate multiple sampling versus multivariate single sample. (A) A multivariate biomarker will require 1 or 2 samples separated by several hours, and by containing information about multiple rhythmic features, the relative level of each of those features can classify the overall circadian timing (Laing et al., 2017). (B) With a univariate biomarker, a time series of points assessing a single feature is collected. Depending on the variability of the feature, “noise” from periodic behaviors or physiologic changes and/or environmental changes may influence any 1 data point or cycle, but multiple cycles of data will provide an accurate assessment of the underlying rhythmic process.

The novel approaches can be broadly classified into (1) circadian phase assessments based on 1 or a few samples of high-dimensional (i.e., multivariate) material, such as the transcriptome or metabolome, and (2) circadian phase assessments based on long-term passive sampling of behavioral, physiological, and/or environmental variables (Fig. 3).

### -Omics-based Assessment of Circadian Phase of Central Pacemaker Using Few Samples

*High dimensional* or *multivariate* refers to samples that contain a large number of variables or features, the constellation of which varies with circadian phase, such that 1 time point suffices to assess any circadian phase. It has long been recognized that a single time point sample of high-dimensional data may contain as much information about circadian phase as a time series of univariate data. The classical example is Linnaeus’s flower clock. Various species of flowers open at different times of day, and any given time of day is characterized by a constellation of open/closed flowers. It turns out that Linnaeus may never have actually planted a flower clock, and the accuracy of flower clocks that were planted by botanical gardens were affected by weather, latitude, and seasonal changes (https://en.wikipedia.org/wiki/Linnaeus%27s_flower_clock). The transcriptome, proteome, and metabolome are high-dimensional data, and a considerable fraction of the thousands of variables in each of these -omics data sets has been shown to be rhythmic in human blood, adipose tissue, skin, and brain (Moller-Levet et al., 2013; Christou et al., 2019; Depner et al., 2018; Ang et al., 2012; Dallmann et al., 2012; Wu et al., 2018).

A main problem in the development of biomarkers from high-dimensional data is the selection of relevant features. Several approaches exist and may be subdivided in those that use a priori knowledge, for example, focus on RNAs from core circadian genes or unbiased approaches (for a discussion, see Laing et al., 2017; Laing et al., 2019b). When unbiased approaches are applied to, for example, the transcriptome, we are exposed to the curse of high dimensionality (e.g., ~20,000 transcripts) and the resultant risk of “overfitting.” When there are many more features than data points to be predicted or described, it is always possible to find a set of features that will fit or describe a particular data set (such as the training data set), but this set of features is much less likely to fit an independent data set (a test set). This problem of overfitting is addressed in machine-learning approaches to feature selection and will not be discussed in detail here (Smith et al., 2014).

A first single-time-point sampling method for the assessment of circadian phase was developed in mice. This was an approach based on a priori knowledge because it used more than 100 time-indicating genes in the liver (Ueda et al., 2004). Subsequently, a single-time-point method for assessment of circadian phase in mice was developed based on plasma metabolites (Minami et al., 2009). The strengths of these single-time-point methods in animals are that the methods were developed while controlling for effects of feeding, sex, age, and the light-dark cycle, as well as that they were validated against gold standard SCN markers such as corticosterone.

In humans, a first blood metabolite-based time table method was developed based on data from 3 participants and validated in 6 participants using plasma cortisol and melatonin as gold standards (Kasukawa et al., 2012). Data were collected under constant routine conditions at the beginning and end of a forced desynchrony protocol, with blood samples drawn every 2 h during a 39-h episode of wakefulness following sleep episodes during the biological night. Using 2 blood samples taken 12 h apart, the reported accuracy was approximately 3 h. The set of metabolites used for the time table construction consisted of 58 rhythmic metabolites, a large fraction of which belonged to metabolism pathways of steroid hormones, such as cortisol.

The first method to use the human blood transcriptome for the prediction of SCN phase was developed based on 329 mRNA samples from 26 participants to build the model, and a validation set of 349 mRNA samples from 27 participants (Laing et al., 2017). The blood samples were collected during a residential stay in a clinical research center while participants were scheduled to a normal sleep-wake cycle, a misplaced sleep-wake cycle, or underwent a period of approximately 40 h of sleep deprivation after a week of sufficient or insufficient sleep (Moller-Levet et al., 2013; Archer et al., 2014). These conditions to some extent mimic conditions such as shift work. Plasma melatonin data were used as a gold standard proxy for SCN phase.

The development of a circadian biomarker based on these data was not based on an initial identification of rhythmic transcripts but used an unbiased machine-learning approach (partial least squares regression) to build a model. Transcripts were quantified by microarray technology using an Agilent platform. For the development of the model, data from all conditions were used, that is, not just baseline but also conditions in which the circadian system was perturbed. The rationale for using samples from various conditions for model development was that in real-world situations, a sample may come from either normal or perturbed conditions, and this will often be unknown. That the effects of sleep timing on putative transcriptome-based biomarkers can be very substantial has been demonstrated in forced desynchrony experiments (Fig. 2B). A sample collected in a shift-work setting may come from a shift worker in whom SCN phase may or may not have adapted to shift work, and it will be unknown whether sleep is or is not displaced relative to SCN phase.

**Figure 4.**
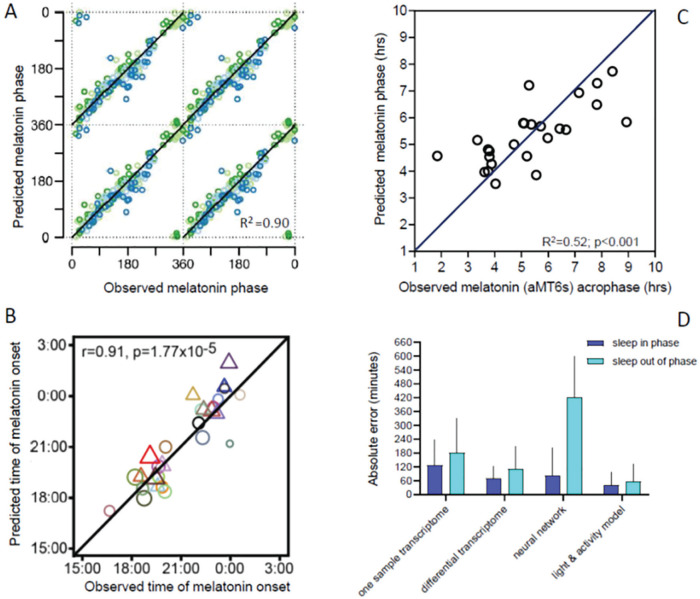
Examples of predictors of circadian melatonin phase and impact of sleeping “out of phase” on accuracy of biomarker prediction. (A) Prediction of plasma melatonin phase from 2 samples taken 12 h apart across the circadian cycle during wakefulness (green symbols), nocturnal sleep (light blue symbols), and misplaced sleep (dark blue circles). From Laing et al. (2017). (B) Prediction of salivary melatonin phase from one sample taken in the afternoon during wakefulness from extreme morning and evening types living on their habitual sleep-wake schedule. Men, triangles; women, circles. The size of the circles indicates the age of the participants. From Wittenbrink et al. (2018). (C) Prediction of urinary 6 sulfatoxy melatonin phase from recordings of activity, light exposure, and a mathematical model for the effects of light in participants living on a nocturnal schedule. From Stone et al. (2019a). (D) Absolute error and its standard deviation of various biomarkers when tested on participants sleeping during the night (in phase) or during the day (out of phase) in either the laboratory or in a shift-work situation. In all cases, the biomarker-predicted phase was compared with a gold standard phase marker (plasma melatonin for the transcriptome predictors; Laing et al., 2017) and urinary 6-sulfatoxy melatonin for the neural network (Stone et al., 2019b) and light model (Stone et al., 2019a). In all cases, accuracy was worse for the out-of-phase condition. Color version of the figure is available online.

This approach identified a set of 100 mRNA abundance features, which was able to predict the melatonin phase in the validation set with an accuracy (SD or error) of 3 h based on 1 sample and 1 h:40 min based on 2 samples taken 12 h apart (82% of samples had an error less than 2 h). The latter approach is essentially a within-subject normalization procedure that improves performance of the method because it removes the large between-subject variation in the blood transcriptome (i.e., the blood transcriptome is to some extent trait-like). The median error (which means that 50% of the samples have an error less than this number) was less than 1 h for the differential model. Importantly, even though the model was developed from samples across a variety of sleep-wake conditions, the accuracy was still less when sleep occurred out of phase (see Fig. 4).

The unbiased approach used in Laing et al. (2017) outperformed time-table methods and ZeitZeiger when applied to the same data set.

Comparing the features identified by each of these methods revealed that many of the features are not directly related to clock genes, but many are related to glucocorticoid signaling pathways.

Hughey and colleagues applied an algorithm (ZeitZeiger) in which a rhythmic spline is fitted to the data (Hughey, 2017) to publicly available data sets collected at the University of Surrey and the University of Pennsylvania (Moller-Levet et al., 2013; Archer et al., 2014; Arnardottir et al., 2014). The algorithm builds a predictor for what is called “circadian time.” Importantly, in this analysis, *circadian time* does not refer to the circadian phase of a sample based on the melatonin phase in that individual but to the time of the sample relative to sunrise or the melatonin phase averaged across individuals. The model was developed on data collected under baseline sleep-wake conditions, and when applied to baseline data, the absolute mean error was 2.1 h. When the model was tested on conditions during which the sleep-wake cycle was displaced, the performance was worse, such that the variability in prediction error increased by 42%, with a median absolute error greater than 3 h. Hughey and colleagues then developed strategies to improve prediction at the individual level by using more than 1 sample per participant, such that using 2 samples taken 8 to 9 h apart resulted in an average improvement in prediction of 0.43 h to approximately 1.67 h. A strength of the ZeitZeiger predictor is that it is based on a small set of 15 genes. In accordance with the results from Laing et al. (2017), only a very few of these 15 genes were core clock genes.

Braun et al. (2018) developed a new algorithm using the University of Surrey and the University of Pennsylvania data sets, as well as a new data set consisting of RNAseq data obtained from healthy participants collected during unperturbed baseline conditions. The authors used a 2-sample approach, and their method requires at least 2 samples, which, for optimal performance, should be drawn 10 to 12 h apart. Unfortunately, the authors did not use a circadian phase marker to validate their method but instead used external clock time (this limitation has been discussed previously; Laing et al., 2019a; Braun et al., 2019). They identified a set consisting of approximately 40 genes, a few of which were core clock genes. The reported median error based on 2 samples was 2 h.

Whereas the Laing et al., Hughey et al., and Braun (CHANGE-18) et al. approaches were based on whole blood, Wittenbrink and colleagues (2018) used monocytes as the source of their transcripts, assuming that rhythmicity would be more robust in this cell type. In addition, the authors argued that because microarray and RNAseq platforms are relatively inaccurate in detecting transcript levels, it may be beneficial to use other platforms for the implementation of circadian phase biomarker sets. Wittenbrink and colleagues took this into consideration when they developed a biomarker for circadian phase using a 2-step process. In the first step, they used ZeitZeiger to identify a set of transcripts quantified by RNAseq. This predictor set was then implemented on a NanoString platform. Their initial search used samples collected in a group of healthy young men in a constant routine protocol, and their validation data set was from a group of individuals with extreme chronotypes living in real-world conditions from whom samples were collected in the morning and afternoon. In both cases, the gold standard proxy for circadian phase was salivary DLMO, which in the validation set varied between approximately 1700 and 0100 h. The authors created 4 predictors: 1 sample–12 genes, 1 sample–2 genes, 2 sample–13 genes, and 2 sample–2 genes. In the validation set, the median absolute error using the Nanostring platform was less than 1 h across all predictors and timing of validation samples (morning, afternoon). The 1 sample–12 genes predictor had an absolute median error of 0.7 h for a morning sample and 0.8 h for afternoon sample. The performance of the 2-sample predictors was similar to the 1-sample predictors (see Fig. 4b). A weakness of this otherwise elegant study is that performance was not assessed under conditions in which sleep was displaced, such as in forced desynchrony, jet lag, or shift work. Thus, we do not yet know to what extent the performance of this method will be affected by the masking effects of sleep, activity, posture, or feeding-fasting, which have been shown to be considerable.

### Long-term Passive Sampling-based Assessment of Circadian Phase Using Activity, Skin Temperature, Heart Rate, Light Exposure, and Other Variables

The desire to assess the SCN phase based on passive monitoring of physiologic and behavioral variables has a long history. Traditionally, these approaches used linear methods in which estimated masking effects were added or subtracted from the observed variables. However, the effects of masking differ across individuals and interact with circadian phase (that is, the masking effects are larger at some circadian phases than others), rendering simple addition/subtraction methods inadequate (Klerman et al., 1999). Recently, more sophisticated approaches in which multiple variables are recorded simultaneously and algorithms are used to predict melatonin phase have been developed using multiple regression or artificial neural network approaches. The various approaches differ primarily with respect to the included variables and required duration of data collection.

### Skin Temperature, Light, and Activity

Whereas core body temperature has long been considered a valuable marker of circadian phase, it is cumbersome to measure by either rectal sensors or thermistor pills that are swallowed. Skin temperature can be measured in a less intrusive manner. The circadian and sleep-wake and activity-dependent regulation of skin temperature has been investigated extensively (Krauchi and Wirz-Justice, 1994). Based on these findings, skin temperature has been evaluated as a source of information about central circadian phase. Kolodyazhniy and colleagues published 2 approaches based on ambulatory skin temperature recordings (from 6 locations), (blue) light recordings from a sensor mounted on glasses, and motion (Kolodyazhniy et al., 2011; Kolodyazhniy et al., 2012). Models were constructed using either multiple regression or artificial neural networks, and the gold standard estimation of SCN phase was based on salivary melatonin collected under constant routine conditions. Participants were healthy but of various chronotypes, with a range of melatonin phases of slightly more than 5 h. Performance of the algorithms was derived from leave-one-participant-out cross-validation. The best model correlated well with melatonin phase (*r* = 0.97), with a SD of the error of only 23 min. In one of the very few instances in which a model developed by one group was tested by another group, Stone et al. (2019b) put this model to the test. Importantly, in this study, the model was tested not only in participants on a normal sleep-wake schedule but also in shift workers, and the gold standard proxy for SCN phase was either DLMO or 6-sulphatoxy melatonin. Whereas in this independent validation, the model performed well under baseline conditions, the performance deteriorated dramatically for assessments in night-shift workers, such that the error was more than 2 h in approximately half of the assessments (see. Fig. 4c). This poor performance persisted even when the model was trained on night-shift data. Performance was even worse when the model was trained on non–shift-work data. The findings of this comprehensive study underscore the necessity to validate any method for circadian phase assessment in situations in which sleep and other masking effects are displaced, not only because these are more challenging conditions for most methods but also because they represent situations in which circadian biomarkers will be used.

### Combining a Mathematical Model for Light with Light Exposure and Activity Data

Although in the studies of Kolodyazhniy et al. and Stone et al., light information was used to build a predictor algorithm, no specific model for how light affects the human circadian pacemaker was employed. Kronauer’s mathematical model for the effects of light (and activity) on the human circadian pacemaker (Jewett et al., 1999; St Hilaire et al., 2007; Kronauer et al., 1982), which is based on the extensive laboratory studies by Czeisler and colleagues (Duffy et al., 1996; Boivin et al., 1996; Czeisler et al., 1989; Jewett et al., 1991; Gronfier et al., 2004; Chang et al., 2012), remains the only quantitative model to date (Duffy and Wright, 2005). It uses light, quantified as lux, as input, and no provision for the spectral composition of light is available. The primary assumption underlying the use of Kronauer’s model to predict circadian phase is that light is the most powerful zeitgeber for the human SCN and that variations in timing and intensity of light exposure, which are in part driven by the timing of sleep and social constraints (Skeldon et al., 2017), are the main determinants of variations in circadian phase. Determinants of phase of entrainment are well understood at a theoretical level (Granada et al., 2013). Phase of entrainment is determined by individual differences in intrinsic period (Wright et al., 2005) and theoretically also by individual differences in light sensitivity. Woelders and colleagues (2017) used Kronauer’s model and reported that DLMO was associated with individual differences in light exposure, such that light + model explained 52% of the variance. When light data and Kronauer’s model were supplemented with activity data, DLMO could be predicted with an SD or error of 1.1 h (i.e., 95% of the predictions had an error of 2.2 h or less). It is noted that although the participants varied with respect to chronotype with a considerable range of measured DLMOs of 9.3 h, they were all sleeping at their habitual bedtimes for the duration of the study. In a study of shift workers on either a diurnal or night schedule, Stone and colleagues (2019a) used a similar approach; that is, they used either a photic-only model or a combination of photic input to Kronauer’s model and activity. The proxy for SCN phase was 6-sulphatoxy melatonin. Performance on the diurnal schedule was comparable and even slightly better than in the Woelders et al. (2017) study. Although performance of the predictors deteriorated somewhat on the night schedule, performance was still rather good, with a SD of 1.39 h and 80% of predictions within 2 h of the observed values (see Fig. 4C). St. Hilaire and colleagues have also used individual light exposure data input to the Kronauer model to predict phase shifts in a simulated shift work study, in which salivary DLMO was assessed before and after a series of night shifts. They found that 85% of the model predictions were within 2 h of the observed DLMO shifts (St. Hilaire and Duffy, personal communication, March 1, 2020).

### Heart Rate

Heart rate and heart rate variability are under control of the autonomic nervous system, which in turn is under SCN control. The masking effect of activity and sleep on heart rate is considerable, but constant routine protocols have demonstrated an endogenous circadian component to heart rate (Viola et al., 2002; Vandewalle et al., 2007). In principle, this implies that ambulatory monitoring of heart rate may provide information on SCN phase provided that masking can be accounted for. Gil and colleagues (2013, 2014) investigated the potential of heart rate measures for circadian phase prediction using only 24 h of data. The proxy for SCN phase was DLMO measured in 11 participants for model development and 19 participants for performance testing. Participants slept at their habitual times, and the range of chronotypes and DLMOs was relatively small (i.e., approximately 3 h). The model was based on an autoregressive moving average with a linear combination of external inputs (e.g., heart rate or light). Using only heart rate as a predictor resulted in an SD of 56 min, similar to the performance of a light input–only model (with light transformed with a power function, as in Kronauer’s model). Combining heart rate and light resulted in performance with an SD of 39 min, and adding activity did not improve performance significantly. Although the performance of these models appears impressive, it should be noted that the range of DLMO was limited, and this model has not been tested under conditions of displaced sleep.

## New Methods to assess Intrinsic Circadian Period

The gold standard assessment of the intrinsic period of the SCN in sighted people is achieved through forced desynchrony of the sleep-wake cycle from the endogenous circadian rhythms by scheduling sleep-wake and the associated dim light-dark cycle to a noncircadian cycle length that is outside the range of entrainment (Klerman et al., 1996; Czeisler et al., 1999; Duffy et al., 2011; Lazar et al., 2013). In totally blind individuals, the intrinsic period can be assessed by repeated phase assessments of 48-h urinary 6-sulphatoxy melatonin while the participants are living in their habitual environment (Hack et al., 2003; Lockley et al., 2015). These experiments have provided important insights into the role of interindividual variation in intrinsic period in entrainment. Several variants of the forced desynchrony protocol have been developed, and a consistent finding across studies is that that in participants without circadian rhythm sleep-wake disorders, the average period is about 24.2 h, with a small standard deviation (Czeisler et al., 1999; Duffy et al., 2011; Micic et al., 2016; Eastman et al., 2017; Hasan et al., 2012). Two alternative period assessment methods have emerged: (1) *in vitro* recording of circadian rhythms in human cell cultures, in which cells are modified to express a luciferase gene under the control of the promoter of a clock gene, and (2) reducing the residual variance of light exposure and mathematical model-derived prediction of circadian phase (DLMO) by optimizing the period parameter of Kronauer’s model at the level of the individual. The latter method has yielded a realistic population average and SD for circadian period, but individual estimates have not been validated against gold standard assessments. Furthermore, the period estimates were based on only 1 circadian phase assessment per participant, and the optimization procedure may therefore not reflect intrinsic period but rather the period that best predicts this single circadian phase (Woelders et al., 2017).

For the *in vitro* circadian period assessment methods, a number of validated studies are available in both blind and sighted individuals. However, in these studies, the average *in vitro* period is longer than that derived from forced desynchrony studies and systematically longer than that derived in the same individuals by melatonin (Pagani et al., 2010; Hasan et al., 2012). In fact, the correlation between the *in vitro* periods and the periods as assessed by urinary 6-sulphatoxy melatonin (in blind individuals) or by plasma melatonin in forced desynchrony studies in sighted people are weak and sometimes not even significant. It thus appears that the current *in vitro* assessments will not be able to accurately assess intrinsic period at the level of the individual.

## New Methods to assess Intrinsic Circadian Amplitude/Circadian Disruption

Changes in the amplitude of overt rhythms such as activity or body temperature are often observed in, for example, aging, and it is also often tacitly assumed that these changes reflect changes in the amplitude of the endogenous circadian components of these variables. As we have previously argued, protocols such as the constant routine are needed to ascertain this (Duffy and Dijk, 2002). However, in general, the concept of circadian amplitude is not well defined, and a simple gold standard measure for circadian amplitude has not been agreed upon. For example, there is substantial variation in melatonin amplitude between individuals and little evidence that those individual differences reflect differences in SCN amplitude. Studies in which a constant routine has been carried out before and after the circadian system has been perturbed have demonstrated that changes in the amplitudes of cortisol, melatonin, and core body temperature in response to an intervention are generally correlated (Jewett et al., 1991; Dijk et al., 2012). This suggests that the amplitudes of these measures may reflect the amplitude of oscillations within the SCN and that it might be possible to develop a metric for circadian amplitude.

New approaches to assess endogenous circadian amplitude are based on multivariate approaches. These methods may not necessarily aim to assess the amplitude of the SCN but may be targeted at assessment of peripheral oscillators. In fact, these methods may not aim to assess the simple construct of amplitude but instead metrics that reflect the robustness or “normality” of circadian processes. The assumption is that under normal conditions, diurnal oscillations in a particular organ or tissue are characterized by a typical progression of expression levels of genes (a tissue-specific temporal program) and in particular genes that are at the core of the generation of circadian rhythmicity. Shilts and colleagues (2018) designed a method to quantify circadian disruption by computing a metric called the clock correlation distance, which is based on the coexpression patterns of 12 clock genes. A larger clock correlation distance indicates less normal, or less robust, rhythmicity. The method was developed on samples from various mouse tissues but then applied to samples from human blood, skin, brain, and *in vitro* cell cultures. The robustness of circadian rhythmicity as detected by this method varied between blood, skin, brain, and *in vitro* cell cultures. The method does not require samples to be labeled with time of day and works best if the entire circadian cycle is covered, but it can perform reasonably well if only part (e.g., one-third) of the circadian cycle is covered. For the method to produce reliable results in humans, it requires approximately 30 samples, and the method cannot be applied to a single sample. One application for this method is in cancer research, and it was indeed shown that this method detects disruption of circadian rhythmicity in tumors.

Vlachou and coworkers (2020) also aimed to develop a method to quantify circadian disruption as well as circadian time with intended application in oncology. Their method, called “time teller,” is based on 10 to 15 genes, requires only 1 sample, and is designed to be used to assess both circadian phase and as circadian dysfunction. As mentioned, these methods to assess circadian disruption are built on the notion that during a normal circadian cycle, at any point in the cycle there is a specific constellation of rhythmic genes being expressed. This assumption can be used to tell time from a single sample but also can be used to detect disruption of circadian organization. Obviously, these methods are very much focused on the local circadian organization of the tissue from which the sample was obtained. Circadian disruption in the periphery does not necessarily imply circadian disruption in the central circadian clock. For example, severe disruption of circadian organization of the transcriptome, including expression of core clock genes, has been observed in the whole-blood transcriptome when sleep was displaced to the daytime, while at the same time, the phase and amplitude of the plasma melatonin rhythm were similar to when sleep occurred at night (Archer et al., 2014).

These observations highlight both the potential of using multivariate data in the periphery to detect circadian disruption or quantify amplitude while at the same time emphasizing the need for careful validation and interpretation of these data. For the assessment of the physiological patency of a local tissue, the cause of the disruption of temporal programs may not be that important. However, in any multioscillator system, local rhythmicity and its disruption may sometimes reflect the disruption of local clocks, disruption of circadian rhythmicity downstream from those local clocks, or disruption of central clocks imposing rhythmicity on those local tissues.

## Concluding Remarks

Wearables and -omics data combined with machine learning and mathematical modeling hold great promise for the development of novel methods to quantify circadian processes in humans. The optimal choice of variables to be collected by wearables, the -omics to be used in “1-sample” methods, or the source of samples (i.e., blood, skin, saliva) remain to be established. This choice of variable(s) will be influenced by the purpose of the biomarker (see Figs. 1 and 2). Until now, much emphasis has been on the development of sophisticated algorithms without clearly stating the purpose of the biomarker. Little effort has been devoted to comparison of these biomarkers to gold standards, validating them in realistic protocols, or defining the required accuracy across use cases. A variety of performance measures have been used in different studies, and reaching a consensus on the performance metrics of biomarkers will facilitate comparison across methods.

It will be useful to evaluate these new methods within a framework that is based on concepts developed in circadian rhythm research together with concepts from the field of biomarker development. Organizations such as the Food and Drug Administration and the National Institutes of Health together with industry partners have provided guidelines for the evidence needed for biomarker qualification and a description of the workflow and decision processes in biomarker development (Leptak et al., 2017). Some of the key concepts that can be applied to circadian biomarkers are “context of use,” which relates to “what question does the biomarker address,” the “biological rationale for use of biomarker,” “independent data sets for qualification,” and “comparison to current standard.”

Circadian concepts such as diurnal versus circadian rhythmicity and masked versus endogenous circadian rhythms will remain useful when evaluating new markers for circadian processes. Likewise, biomarker concepts such as robustness, reliability, sensitivity, and specificity should be formalized for our field and applied to any novel method designed for quantifying circadian rhythms. It may be unrealistic to expect that a particular biomarker for, for example, circadian phase, is universally robust, that is, it can be applied in a wide range of situations and populations, and it may be that situation-, population-, or diagnosis-specific approaches can be developed. However, in practice, the precise situation, population, or diagnosis will be often unknown. Therefore, for these novel, be it universal or specific, biomarkers to be useful, they should be validated in controlled laboratory settings where the rest-activity schedules of participants are manipulated, tested in men and women of all ages, tested in large groups of normal individuals in real-life situations, and tested in patient populations, particularly those patient populations in which these methods will be applied.
